# Presynaptic nanodomains: a tale of two synapses

**DOI:** 10.3389/fncel.2014.00455

**Published:** 2015-01-26

**Authors:** Lu-Yang Wang, George J. Augustine

**Affiliations:** ^1^Program in Neurosciences and Mental Health, SickKids Research InstituteToronto, Canada; ^2^Department of Physiology, University of TorontoToronto, Canada; ^3^Lee Kong Chian School of Medicine, Nanyang Technological UniversitySingapore, Singapore; ^4^Institute of Molecular and Cell BiologySingapore, Singapore; ^5^Center for Functional Connectomics, Korea Institute of Science and TechnologySeoul, South Korea; ^6^Marine Biological LaboratoryWoods Hole, MA, USA

**Keywords:** neurotransmitter release, calcium signaling, calcium channels, presynaptic terminals, synaptic vesicle trafficking

## Abstract

Here we summarize the evidence from two “giant” presynaptic terminals—the squid giant synapse and the mammalian calyx of Held—supporting the involvement of nanodomain calcium signals in triggering of neurotransmitter release. At the squid synapse, there are three main lines of experimental evidence for nanodomain signaling. First, changing the size of the unitary calcium channel current by altering external calcium concentration causes a non-linear change in transmitter release, while changing the number of open channels by broadening the presynaptic action potential causes a linear change in release. Second, low-affinity calcium indicators, calcium chelators, and uncaging of calcium all suggest that presynaptic calcium concentrations are as high as hundreds of micromolar, which is more compatible with a nanodomain type of calcium signal. Finally, neurotransmitter release is much less affected by the slow calcium chelator, ethylene glycol tetraacetic acid (EGTA), in comparison to the rapid chelator 1,2-bis(o-aminophenoxy)ethane-N,N,N’,N’-tetraacetic acid (BAPTA). Similarly, as the calyx of Held synapse matures, EGTA becomes less effective in attenuating transmitter release while the number of calcium channels required to trigger a single fusion event declines. This suggests a developmental transformation of microdomain to nanodomain coupling between calcium channels and transmitter release. Calcium imaging and uncaging experiments, in combination with simulations of calcium diffusion, indicate the peak calcium concentration seen by presynaptic calcium sensors reaches at least tens of micromolar at the calyx of Held. Taken together, data from these provide a compelling argument that nanodomain calcium signaling gates very rapid transmitter release.

## Introduction to the calcium domain concept

The concept of local “domains” of intracellular calcium concentration developed from mathematical models of the diffusion of Ca ions (Ca^2+^) away from the mouth of open calcium channels. The first published model, by Chad and Eckert (1984), laid out several key features of this concept. This paper proposed that opening of individual Ca^2+^ channels could form discrete spatial domains where Ca^2+^ concentration is very high locally—within nanometers of a single open Ca^2+^ channel—but drops off steeply with distance away from the channel. In addition, increasingly large depolarizations of the membrane potential should have dual effects: increasing the number of such domains, by increasing the number of open Ca^2+^ channels, yet reducing the magnitude of the local Ca^2+^ concentration change within a domain because of the reduced unitary current through an open channel.

Simon and Llinás (1985) independently developed the Ca^2+^ domain concept and considered the possible role of such domains in triggering of neurotransmitter release from presynaptic terminals. In addition to confirming the deductions of Chad and Eckert (1984) regarding the basic properties of Ca^2+^ domains, they further concluded that these domains usually do not overlap appreciably for the case of squid presynaptic terminals. When many Ca^2+^ channels are opened by large, prolonged depolarizations, there can be a small degree of spatial overlap due to summation of Ca^2+^ from adjacent open channels. The modeling studies of Zucker and Fogelson (1986) reached a similar conclusion. Notably, Simon and Llinás (1985) concluded that an endogenous Ca^2+^ buffer could eliminate summation of Ca^2+^ signals associated with neighboring domains, but should have minimal effect on the peak Ca^2+^ concentration change occurring in the immediate vicinity of an open channel.

At its simplest, diffusion of Ca^2+^ from an open channel can be described by the following equation (Smith and Augustine, 1988):

(1) $$\left[{\text{Ca}}^{\text{2+}}\right]\left(x,t\right)=\frac{J_{Ca}}{2\pi D_{x}}erfc\frac{x}{2\sqrt{Dt}}$$

where *x* is distance from the channel, *t* is time, *J_Ca_* is the flux of Ca^2+^ through a single open channel, *D* is the diffusion coefficient for Ca^2+^ and *erfc* is the complementary error function. This equation predicts that (1) while the channel is open, there will be an inverse relationship between Ca^2+^ concentration and distance from the channel; and (2) Ca^2+^ diffusion time will increase with the square of distance. To aid comprehension of these relationships, we provide “rule of thumb” Ca^2+^ diffusion parameters in Table 1. These parameters are to be taken as order-of-magnitude approximations because their precise values will depend on the value chosen for *D*, as well as the unitary current flowing through the open Ca^2+^ channel and the properties of cellular Ca^2+^ buffering. More complete mathematical treatments can be found elsewhere, for example in Smith and Augustine (1988), Naraghi and Neher (1997) and Neher (1998), as well as in the pioneering domain papers described above.

**Table 1:** **“Rule of thumb” relationships for Ca^2+^ diffusion within domains**.

Distance	[Ca^2+^] (~1/***x***)	Time (~*x*^2^)
10 nm	100 μM	1 sec
100 nm	10 M	100 sec
1000 nm	1 M	10000 sec

**Figure 1:**
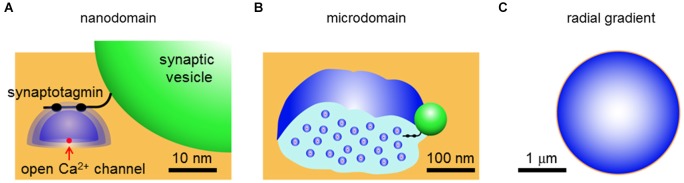
**Three types of local Ca^2+^ signaling. (A)** Nanodomains arise from Ca^2+^ (blue) diffusing away from a single open Ca^2+^ channel (red) in the presynaptic plasma membrane (orange). The green spherical structure is a synaptic vesicle and a synaptotagmin-like Ca^2+^ sensor is shown protruding from the surface of the vesicle. **(B)** Microdomains arise from spatial overlap of local Ca^2+^ signals contributed by multiple open Ca^2+^ channels within a micrometer of each other. **(C)** Terminal-wide radial gradients of Ca^2+^ arise from long-distance diffusion of Ca^2+^ from channels at the plasma membrane. Derived from illustrations in Schweizer et al. (1995) and Augustine et al. (2003).

As mentioned above, it is possible that Ca^2+^ signals from adjacent open Ca^2+^ channels could overlap under some conditions. The degree of overlap leads to several qualitatively different forms of local Ca^2+^ signaling that are depicted in Figure 1. If there is no spatial overlap between the local Ca^2+^ domains, relative to the position of the relevant Ca^2+^ sensor, then a nanodomain occurs (Figure 1A). This type of signal is called a nanodomain because Ca^2+^ signaling will occur over dimensions of nanometers (Kasai, 1993; Schweizer et al., 1995; Augustine et al., 2003). If there is some degree of spatial overlap between neighboring domains and the Ca^2+^ sensor receives contributions from domains summing over a distance of approximately a micrometer, then this is microdomain-style Ca^2+^ signaling (Figure 1B). Finally, if the sensor receives Ca^2+^ ions diffusing over distances greater than a micrometer, due to summation of microdomains, then a radial gradient style of Ca^2+^ signaling is said to occur (Figure 1C). The differences in the spatial range of these different modes of Ca^2+^ signaling necessarily are associated with differences in the speed of Ca^2+^ triggering of biological processes, as indicated by the *t*-term in equation (1) and in the right column of Table 1.

From a physical perspective, there is no question that local domains of Ca^2+^ signaling occur in the immediate vicinity of an open Ca^2+^ channel. The question addressed in this review is how relevant such domain signaling is for release of neurotransmitters from presynaptic terminals. We will compare Ca^2+^ signaling at two presynaptic terminals whose large size makes them particularly amenable to experimental analysis: the squid giant synapse and the mammalian calyx of Held. From results obtained at these synapses, as well as selected results from other synapses, we will develop the argument that nanodomain Ca^2+^ signaling is prevalent at presynaptic terminals that rapidly release neurotransmitters, though microdomain signaling also has been established for some types of presynaptic terminals. Radial gradients are likely to be important only for presynaptic terminals that slowly release neuropeptides or for slower modulatory presynaptic actions of Ca^2+^ within fast-acting synapses, for example Ca^2+^-dependent forms of synaptic plasticity.

## Evidence for nanodomains at the squid giant synapse

Virtually all of the early experimental tests of the role of Ca^2+^ domains in triggering neurotransmitter release were performed at the squid giant synapse. As a result, work at this synapse established most of the experimental paradigms that have been used to probe local Ca^2+^ signaling in presynaptic terminals. In this section we will describe some of these key experiments.

The first clues regarding the possible role of presynaptic domain signaling came from analysis of the kinetics of transmitter release. The minimum synaptic delay between a presynaptic Ca^2+^ “tail” current (following a depolarization to a very positive membrane potential) and the resultant postsynaptic response is very brief, on the order of hundreds of microseconds (Llinás et al., 1981; Augustine et al., 1985). Given the “rule of thumb” relationships for local Ca^2+^ signaling described above (Table 1), this indicates that Ca^2+^ must diffuse on the order of 100 nm or less before binding to the Ca^2+^ sensor for neurotransmitter release. This is consistent with a nanodomain type of presynaptic Ca^2+^ signal. Further, Simon and Llinás (1985) indicated that the relationship between presynaptic Ca^2+^ current and postsynaptic response could be explained by a model where domains do not overlap appreciably, i.e., nanodomain signaling.

While these initial observations provided indirect support for the role of nanodomain Ca^2+^ signals in triggering transmitter release at the squid giant synapse, they were open to alternative interpretations. However, several additional experiments have greatly bolstered acceptance of this notion. This experimental evidence can be grouped into three categories.

### Manipulations of domain size and number

Since the classic work of Dodge and Rahamimoff (1967), it has been known that Ca^2+^ acts “cooperatively”, meaning that there is a high-order relationship between extracellular Ca^2+^ concentration and the amount of neurotransmitter release evoked by presynaptic action potentials. Cooperative triggering of transmitter release can be used as a tool to probe the spatial organization of presynaptic Ca^2+^ signaling.

**Figure 2:**
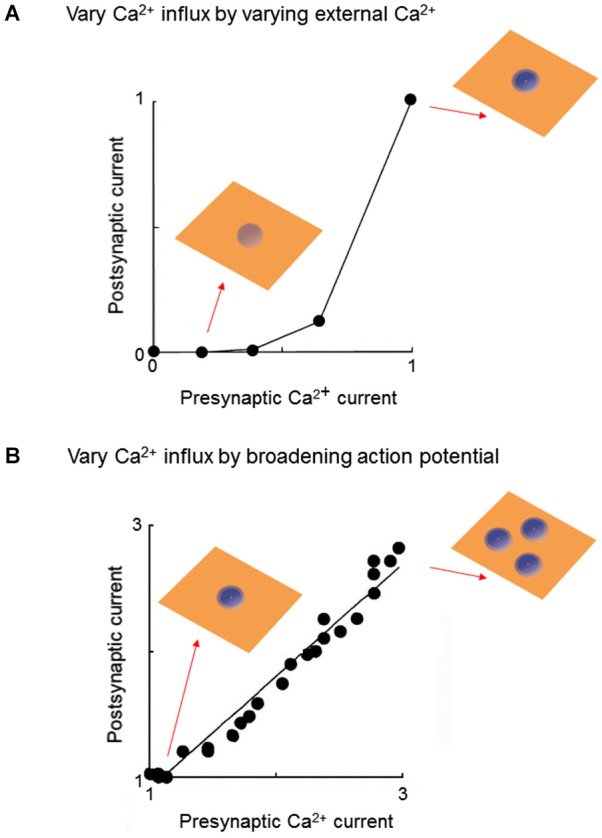
**Experimental strategies for probing local Ca^2+^ signaling during neurotransmitter release. (A)** At a voltage-clamped squid giant synapse, varying external Ca^2+^ concentration changes presynaptic Ca^2+^ influx by keeping number of Ca^2+^ domains constant, while changing the amount of Ca^2+^ within each domain (insets). Under these conditions, the relationship between presynaptic Ca^2+^ current and amount of transmitter release, assessed by amplitude of the postsynaptic current, is a highly non-linear power function. Both presynaptic and postsynaptic responses are normalized to the maximum values measured at the highest external Ca^2+^ concentration. Data replotted from Augustine and Charlton (1986). **(B)** Pharmacologically broadening the presynaptic action potential at the squid giant synapse varies Ca^2+^ influx by opening additional Ca^2+^ channels, thereby increasing the number of Ca^2+^ domains (insets). Under these conditions, the relationship between presynaptic Ca^2+^ current and amount of transmitter release is a linear function. Both presynaptic and postsynaptic responses are normalized to the values measured under control conditions (narrowest presynaptic action potential). Data replotted from Augustine et al. (1991).

Voltage clamp experiments at the squid giant synapse allowed the first direct measurement of Ca^2+^ entry into a presynaptic terminal and established that this cooperativity arises from a non-linear relationship between Ca^2+^ entry and the resultant release of neurotransmitters. Initially Ca^2+^ entry was varied by depolarizing the presynaptic membrane potential to different levels, thereby varying the number of open Ca^2+^ channels. While an initial account proposed a linear relationship between Ca^2+^ entry and transmitter release (Llinás et al., 1981), a follow-up study that optimized spatial control of presynaptic membrane potential indicated a third- or fourth-power relationship (Augustine et al., 1985). Because depolarizing to different membrane potentials changes the number of Ca^2+^ domains, as well as the Ca^2+^ concentration within each domain, these results are not easily interpreted in terms of local Ca^2+^ signals. A follow-up study by Augustine and Charlton (1986) circumvented this problem by depolarizing to a constant presynaptic membrane potential, to open a constant number of Ca^2+^ channels, while varying Ca^2+^ entry by changing external Ca^2+^ concentration. Under such conditions, an approximate fourth-power relationship between Ca^2+^ entry and transmitter release was observed (Figure 2A). Given that the number of Ca^2+^ domains will be constant under these conditions (Figure 2A insets), this result reveals that cooperativity occurs within the domains.

A strikingly different result is obtained when Ca^2+^ entry is varied by altering the duration of the presynaptic action potential. Blockade of potassium channels prolongs the duration of the presynaptic action potential and thereby increases Ca^2+^ entry into the terminal by increasing the number of open Ca^2+^ channels (Klein and Kandel, 1980). Under such conditions, a linear relationship between action potential duration and postsynaptic response was observed (Augustine, 1990). Although Ca influx was not measured directly, the known gating properties of the squid presynaptic Ca^2+^ channels allowed calculation of the presynaptic Ca^2+^ current and indicated a linear relationship between action potential duration and Ca^2+^ current magnitude (Augustine, 1990). Thus, there is a linear relationship between Ca^2+^ current and transmitter release when Ca^2+^ influx is varied by changing the number of open Ca^2+^ channels (Figure 2B; Augustine et al., 1991).

**Figure 3:**
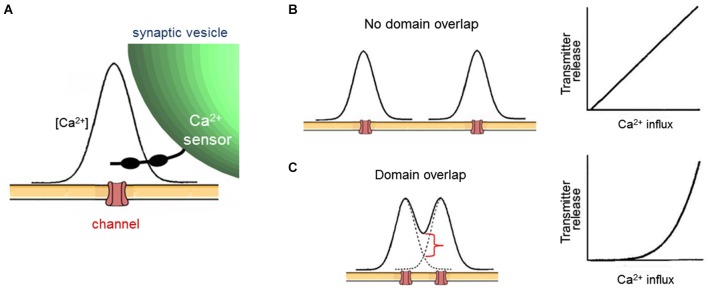
**Evaluating local Ca^2+^ signaling during broadening of presynaptic action potentials. (A)** Diagram of spatial distribution of Ca^2+^ associated with a single open Ca^2+^ channel. **(B)** If open Ca^2+^ channels are sufficiently far apart, then Ca^2+^ signals associated with each channel will not overlap, creating a nanodomain arrangement (left). In this case, broader presynaptic action potentials will increase the number of discrete domains, leading to a proportional increase in transmitter release and a linear relationship between Ca^2+^ influx and release (right). **(C)** If open Ca^2+^ channels are close together, then Ca^2+^ signals associated with each channel will overlap, causing an increase in Ca^2+^ concentration (red bracket) within the local Ca^2+^ signal associated with each channel (left). This creates a microdomain Ca^2+^ signal. In this case, broader presynaptic action potentials will increase the Ca^2+^ concentration within each domain, leading to a supralinear increase in transmitter release due to the cooperative triggering of transmitter release by Ca^2+^. This spatial summation will yield a supralinear relationship between Ca^2+^ influx and release (right) when presynaptic action potentials are broadened. After Augustine et al. (1991).

Given that cooperativity occurs within a domain (Figure 2A), the fact that increasing the number of domains causes a linear increase in transmitter release (Figure 2B) leads to the conclusion that domains do not overlap during a presynaptic action potential. The logic behind this deduction is shown in Figure 3. In brief, if domains do not overlap then transmitter release will increase linearly according to the number of Ca^2+^ channels opened (Figures 3A,B). If domains overlap, then release will increase supralinearly due to summation of Ca^2+^ from neighboring domains, as well as the cooperative action of Ca^2+^ within a domain (Figure 3C). The results of Figure 2B are consistent with the expectations if domains do not overlap (Figure 3B, right). Thus, domains do not overlap during a presynaptic action potential at the squid giant synapse. Similar lines of experimentation have been used to probe the spatial dimensions of Ca^2+^ signaling in other presynaptic terminals (e.g., Wu and Saggau, 1994; Mintz et al., 1995).

### Measurements of local calcium concentration

If the Ca^2+^ sensor for neurotransmitter release is located within nanometers of an open Ca^2+^ channel, as postulated by a nanodomain coupling scheme, then the local Ca^2+^ concentration experienced by this sensor should be in the range of tens to hundreds of micromolar (Table 1). Two types of experimental paradigms have tested this premise at the squid giant synapse.

The first experiment compared the ability of Ca^2+^ chelators of differing affinities to block transmitter release. For this purpose, analogs of the Ca^2+^ chelator 1,2-bis(o-aminophenoxy)ethane-N,N,N’,N’-tetraacetic acid (BAPTA) were microinjected into the giant presynaptic terminal. A complex relationship between Ca^2+^ affinity and chelator efficacy was observed: the most potent inhibitor of transmitter release was dibromoBAPTA, a chelator with intermediate Ca^2+^ affinity (4.9 µM), while chelators with lower or higher affinities were less potent (Adler et al., 1991). A numerical model could predict such results only by assuming that the presynaptic Ca^2+^ sensor for transmitter release encounters Ca^2+^ levels on the order of 100 µM or higher (Augustine et al., 1991). This prediction is consistent with the results of experiments employing photolysis of a caged Ca^2+^ chelator; even raising presynaptic Ca^2+^ concentration to 50 µM produced rates of transmitter release lower than those evoked by a presynaptic action potential (Hsu et al., 1996). Together, these results are compatible with the predictions of a nanodomain coupling model.

The second experiment imaged presynaptic Ca^2+^ concentration during transmitter release. For this purpose, Llinás et al. (1992) used a version of the bioluminescent Ca^2+^-binding protein, aequorin, that was engineered to have an especially low affinity for Ca^2+^. This indicator protein, called n-Aequorin-J, only emits light at Ca^2+^ concentrations in the micromolar range or higher, with the amount of light emission increasing steeply as a function of Ca^2+^ concentration. Based on the amount of bioluminescence emitted by n-Aequorin-J injected into the squid giant presynaptic terminal, Llinás et al. (1992) concluded that presynaptic Ca^2+^ concentration reached 200–300 µM during transmitter release. Further, their measurements indicated that the regions of high Ca^2+^ concentration were quite localized, being a fraction of a square micrometer in area. While there are caveats regarding calibration of this indicator, as well as the spatial resolution of the Ca^2+^ imaging method, the results are compatible with the nanodomain coupling model.

In sum, estimates provided by two complementary experimental approaches indicate that Ca^2+^ concentration rises on the order of hundreds of micromolar in the squid giant presynaptic terminal during transmitter release. Such results favor nanodomain coupling of Ca^2+^ channels to transmitter release at this synapse.

### Differential effects of calcium chelators

The final experimental paradigm—and the one that has proven most useful for probing local Ca^2+^ signaling in presynaptic terminals—is based on comparison of Ca^2+^ chelators with different rates of Ca^2+^ binding (Adler et al., 1991). BAPTA binds Ca^2+^ very rapidly, with its on rate being diffusion-limited, while ethylene glycol tetraacetic acid (EGTA) binds Ca^2+^ more slowly because it is protonated at physiological pH and the bound proton must dissociate before Ca^2+^ can bind. This greatly slows the binding of Ca^2+^ to EGTA in comparison to BAPTA, even though the equilibrium affinity of these two chelators is roughly similar (Augustine et al., 1991). The slow binding of Ca^2+^ allows EGTA to serve as a high-pass temporal (and spatial) filter for Ca^2+^.

**Figure 4:**
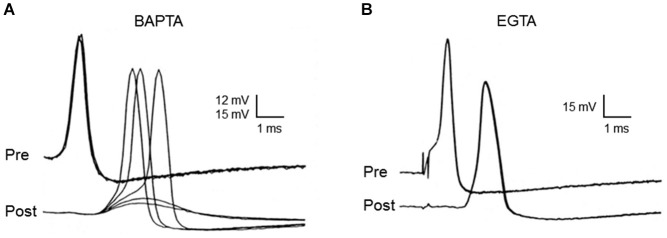
**Differential effects of EGTA and BAPTA on transmitter release at the squid giant synapse**. While presynaptic microinjection of the rapid Ca^2+^ chelator, BAPTA, blocks transmitter release **(A)**, microinjection of the slower chelator, EGTA, has no effect. **(B)** Each family of traces shows five superimposed presynaptic action potentials (Pre), as well as the postsynaptic responses (Post) that they evoke, during the course of chelator injection. While BAPTA reduces synaptic transmission to the point that postsynaptic responses are subthreshold, EGTA has no effect on postsynaptic response strength. Traces originally from Adler et al. (1991).

Neurotransmitter release evoked by presynaptic action potentials is completely inhibited when BAPTA is microinjected into the squid giant presynaptic terminal (Figure 4A). In contrast, neurotransmitter release is unaffected when EGTA is injected even at concentrations as high as 80 mM (Figure 4B). Injection of EGTA is capable of buffering slower Ca^2+^ signaling processes, such as Ca^2+^ triggering of synaptic augmentation (Swandulla et al., 1991).

The interpretation of these results is that differences in the Ca^2+^ binding rates of these two chelators are responsible for their differential effects on neurotransmitter release. Thus, BAPTA can block transmitter release because it binds Ca^2+^ more rapidly than the Ca^2+^ sensor for transmitter release, while EGTA fails to block transmitter release because it binds Ca^2+^ slower than this sensor. Under conditions appropriate for the squid giant synapse, 80 mM EGTA will bind Ca^2+^ with a time constant of approximately 50 µsec, while the same concentration of BAPTA requires only a fraction of a microsecond (Augustine et al., 1991). Thus, the Ca^2+^ sensor for transmitter release must bind Ca^2+^ in more than 1 µsec and in less than 50 µsec. Knowledge of this temporal constraint has been useful in establishing synaptotagmin as the Ca^2+^ sensor for transmitter release (Hui et al., 2005). In regard to the spatial dimensions of Ca^2+^ signaling during transmitter release, a time window of tens of microseconds means that Ca^2+^ diffuses no more than tens of nanometers prior to triggering transmitter release (Table 1). Thus, a process that is blocked by BAPTA but not by EGTA must be mediated by nanodomain Ca^2+^ signaling and this is the case for transmitter release at the squid giant synapse.

In summary, a variety of different experimental paradigms all point to the conclusion that transmitter release at the squid giant synapse is caused by nanodomain-style coupling of presynaptic Ca^2+^ channels to the presynaptic Ca^2+^ sensor, which presumably is synaptotagmin. In the next section we will see how these and other paradigms have been used to probe the spatial dimensions of presynaptic Ca^2+^ signaling at a different “giant” synapse, the mammalian calyx of Held.

## Evidence for nanodomains at the calyx of Held synapse

The giant calyx of Held synapse has been one of the most prominent preparations developed over the past two decades for analysis of synaptic transmission in the mammalian brain (Forsythe, 1994; Borst et al., 1995). Unfortunately, there is conflicting evidence about whether microdomain or nanodomain Ca^2+^ signaling mediates transmitter release at this synapse (Borst and Sakmann, 1996; Fedchyshyn and Wang, 2005). In this section we will survey this evidence and suggest that the conflict can be resolved by concluding that the spatial range of presynaptic Ca^2+^ signaling changes over the course of maturation of this synapse.

**Figure 5:**
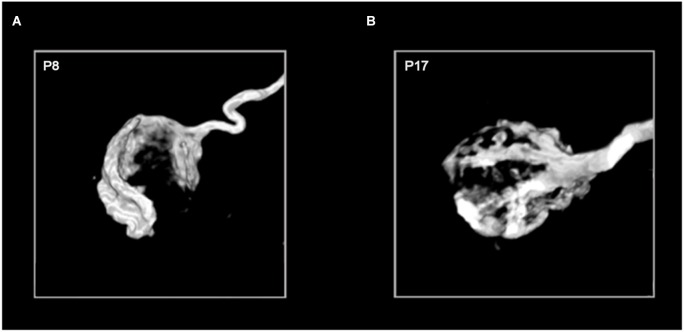
**Age-dependent morphological remodeling of the calyx of Held**. Images of the 3-dimensional structure of P8 **(A)** and P17 **(B)** calyces filled with the fluorescent marker biotinylated dextran amine (courtesy of G. Grande). The diameter of each calyx sphere is approximately 15–20 micrometers.

The calyx of Held synapse undergoes tremendous morphological remodeling in the course of its development, with its presynaptic terminal transforming from a spoon- or club-like structure with thin filopodia before the onset of hearing (P11–12) to a highly digitated structure with stalks and swellings containing a total of 500–800 active zones at maturity (>P16; Figure 5). Such structural transformations are thought to be important for supporting release and replenishment of synaptic vesicles (SVs), as well as facilitating clearance of neurotransmitter to alleviate postsynaptic receptor desensitization during high-frequency transmission (Trussell, 1999; von Gersdorff and Borst, 2002). In practice, calyces become less visible in brainstem slices for patch-clamping as myelination progresses during maturation, meaning that most early studies on the calyx of Held synapse focused on immature synapses (e.g., P8–11).

### Evidence against nanodomain coupling

Using recordings of presynaptic Ca^2+^ currents (*I_Ca_*) and excitatory postsynaptic currents (*I_EPSC_*) from young (P8–11) calyx of Held synapses, Borst and Sakmann (1996) first demonstrated that the amplitude of excitatory postsynaptic currents (EPSCs) follows the fourth power of presynaptic Ca^2+^ influx, similar to the squid giant synapse (Figure 2A). However, unlike the squid giant synapse, injection of EGTA at a concentration as low as 1 mM attenuated EPSCs by about 50%, leading to the conclusion that Ca^2+^ influx from many open channels (specifically >60) triggers fusion of a single synaptic vesicle. Furthermore, N-, P/Q, and R-type Ca^2+^ channels were found to jointly mediate transmitter release at this synapse, with P/Q-type Ca^2+^ channels being the most prominent subtype (Forsythe et al., 1998; Iwasaki and Takahashi, 1998; Wu et al., 1998, 1999). By photolysis of caged Ca^2+^ and computer simulations with Ca^2+^ buffer models (Bollmann et al., 2000; Schneggenburger and Neher, 2000), it was estimated that the peak Ca^2+^ concentration at Ca^2+^ sensors on SV is as low as 10 µM, an order of magnitude lower than that deduced for the squid giant synapse. These results are in striking contrast to what would be expected for nanodomain coupling, arguing in favor of a microdomain mode of Ca^2+^ signaling at the immature calyx of Held synapse (Meinrenken et al., 2002).

### Evidence for nanodomain coupling

In parallel with morphological remodeling at the developing calyx of Held, presynaptic action potentials undergo a dramatic shortening in their duration (Taschenberger and von Gersdorff, 2000), which would reduce the number of activated Ca^2+^ channels and thereby attenuate Ca^2+^ influx. Given that the relationship between *I_Ca_* and *I_EPSC_* is a power function

(2) $$I_{EPSC}\propto {\left[I_{Ca}\right]}^{n}$$

where *n* denotes Ca^2+^ cooperativity (typically a value of 3–5; Augustine et al., 1985), spike narrowing would lead to a significant reduction in *I_EPSC_*. In fact, quantal output is increased in older mice (Joshi and Wang, 2002) or maintained in rats (Iwasaki and Takahashi, 2001). This created a paradox: how could the fewer Ca^2+^ channels recruited by narrower action potentials during development somehow become more effective in triggering transmitter release (Yang and Wang, 2006; Kochubey et al., 2009; Leão and von Gersdorff, 2009)?

Two possible mechanisms have been considered: (1) the spatial coupling between open Ca^2+^ channels and Ca^2+^ sensors tightens to yield nanodomain signaling; and/or (2) the Ca^2+^ sensor (e.g., synaptotagmin) becomes more sensitive to Ca^2+^ to detect smaller rises in presynaptic Ca^2+^ concentration. To test the first possibility, Fedchyshyn and Wang (2005) injected EGTA (10 mM) into developing calyx of Held synapses and found that while this slow Ca^2+^ buffer potently attenuates transmitter release in immature terminals (P8–12 mice), as previously reported (Borst and Sakmann, 1996), EGTA had surprisingly little effect at mature synapses (P16–18). This indicates that the distance between Ca^2+^ channels and Ca^2+^ sensors shortens over the course of development. A similar conclusion was reached by using capacitance measurements of exocytosis under different Ca^2+^ buffering conditions (Leão and von Gersdorff, 2009). By using a depolarizing paradigm that recruits an increasing number of Ca^2+^ channels without changing the kinetics of *I_Ca_* or the driving force for Ca^2+^, Fedchyshyn and Wang (2005) further demonstrated that Ca^2+^ cooperativity values (*n*) were significantly higher in immature synapses than in mature synapses. Based on the logic depicted in Figure 3, this suggests that the number of Ca^2+^ channels required for triggering release of a synaptic vesicle decreases. It has also been shown that while transmitter release at P8–12 synapses requires both N- and P/Q-type Ca^2+^ channels, only P/Q-type Ca^2+^ channels are involved at mature synapses. This suggests that nanodomains contain specific type of Ca^2+^ channels as part of their molecular architecture.

To test the second possibility, Ca^2+^ sensor sensitivity was measured in the developing calyx of Held synapse via simultaneous Ca^2+^ imaging and flash photolysis of caged Ca^2+^ chelators (Wang et al., 2008; Kochubey et al., 2009). Flash photolysis of caged Ca^2+^ has the advantage of bypassing Ca^2+^ entry through Ca^2+^ channels, instead directly triggering release by increasing intracellular Ca^2+^ concentration ([Ca^2+^]_i_) in a spatially homogeneous manner. Surprisingly, Ca^2+^ sensor sensitivity decreases over development (e.g., *K*_D_ values rise from ~80 µM for P9–P11 vs. ~120 µM for P16–P19 synapses) while cooperativity (*n*) remains the same. Taken together, these studies led to the conclusion that the spatial coupling between Ca^2+^ channels and Ca^2+^ sensors switches from microdomain to nanodomain signaling.

**Figure 6:**
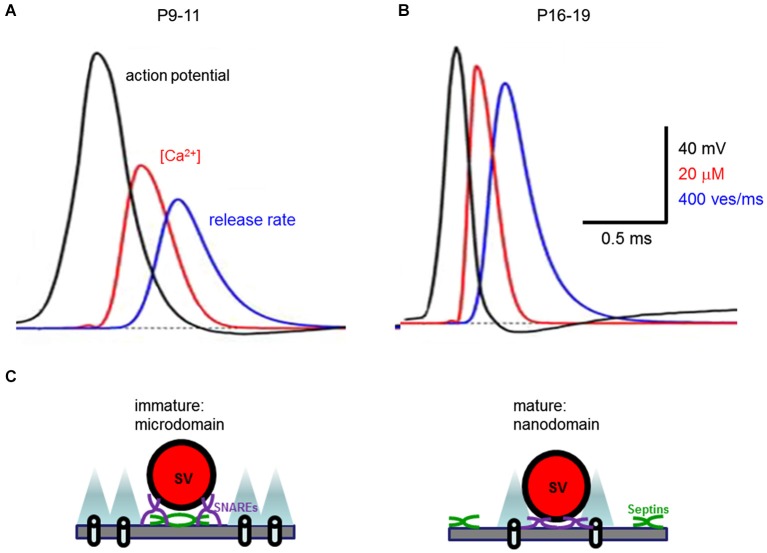
**Developmental change in presynaptic Ca^2+^ signaling at the calyx of Held synapse. (A,B)** Developmental tightening of coupling between Ca^2+^ channels and synaptic vesicles boosts the local [Ca^2+^]_i_ transient seen by the Ca^2+^ sensor (red traces) and the rate of transmitter release (blue traces) as action potentials (black traces) narrow (from Wang et al., 2008). **(C)** Transformation from microdomain to nanodomain coupling of Ca^2+^ channels to transmitter release. Note that nanodomains are associated with shorter distance between Ca^2+^ channels and synaptic vesicles (SV) both in the plane of the presynaptic plasma membrane, as well as orthogonal to this membrane. This spatial reorganization of Ca^2+^ signaling is associated with translocation of septins (green) away from the active zone during development.

Such changes can account for the upregulation of transmitter release over the course of development of the calyx of Held synapse. Computer simulations with a linearized buffered Ca^2+^ diffusion model and a five-site kinetic model of transmitter release (Naraghi and Neher, 1997; Schneggenburger and Neher, 2000) deduced that during development the peak Ca^2+^ concentration seen by a Ca^2+^ sensor during an action potential increases from 35 to 56 µM (Figures 6A,B). This is a consequence of tightened spatial coupling between Ca^2+^ channels and sensors; the modeling results indicate that the microdomain signaling during an action potential in immature synapses results from 12 Ca^2+^ channels with 50% open probability and 61 nm separation, while the nanodomain coupling at mature synapses results from 9 Ca^2+^ channels with 35% open probability and 23 nm spacing (Figure 6C; Wang et al., 2008, 2009). This change in the spatial dimensions of presynaptic Ca^2+^ signaling nearly doubles the release rate and enables the peak of the local [Ca^2+^]_i_ transient to occur ~410 µs earlier in mature synapses than in immature synapses (Figures 6A,B). Although direct evidence for the topographic organization of Ca^2+^ channels at the developing calyx remains elusive, these simulation results provide a theoretical framework for envisaging how a developmental transition from microdomain to nanodomain coupling can recapitulate a number of experimental observations, including an explanation for how narrower action potentials become more effective in triggering transmitter release (Taschenberger and von Gersdorff, 2000).

## Evidence for nanodomain coupling at other synapses

The squid giant synapse and calyx of Held synapse are often considered to be specialized synapses because of their extraordinary size, speed and strength of synaptic transmission. Hence nanodomain coupling could be a feature unique to such high-fidelity, excitatory “relay” synapses. However, this clearly is not the case because a number of synapses have now been shown to release neurotransmitters via nanodomain coupling.

In line with experiments suggesting nanodomain coupling of Ca^2+^ influx to transmitter release at the squid giant synapse and calyx of Held, early studies indicated that Ca^2+^ channels are tightly coupled to Ca^2+^ sensors to enable highly efficient transmission at neuromuscular synapses (Yoshikami et al., 1989; Delaney et al., 1991). In some cases, opening of a single Ca^2+^ channel is sufficient for triggering of fusion events at the active zones of chick ciliary ganglion synapses (Stanley, 1993; Gentile and Stanley, 2005).

Initial studies in mammalian synapses appeared to argue in favor of microdomain coupling (Luebke et al., 1993; Takahashi and Momiyama, 1993; Wheeler et al., 1994; Wu and Saggau, 1994; Mintz et al., 1995). These studies combined Ca^2+^ imaging in presynaptic terminals with patch-clamp recordings from postsynaptic neurons and treatment with subtype-specific Ca^2+^ channel blockers. The results collectively showed that not only are multiple Ca^2+^ channels involved in presynaptic Ca^2+^ signaling but also that different subtypes of Ca^2+^ channels can mediate transmitter release at a synapse, leading to the idea that opening of many adjacent Ca^2+^ channels forms microdomains.

It should be noted that the conditions employed in these different experiments differ in many ways. For example, the fraction of Ca^2+^ channels activated by an action potential differ, ranging from ~10% at the squid giant synapse to >70% at some mammalian central synapses. In addition, extracellular Ca^2+^ concentrations ranged from 11 mM for the squid synapse to 1–2 mM for mammalian central synapses and experimental temperatures varied from 15 to 37^°^C. All of these variables profoundly influence the number of Ca^2+^ channels opened by an action potential, as well as the driving force on Ca^2+^ and the kinetics of Ca^2+^ diffusion within nerve terminals, thereby affecting the coupling of Ca^2+^ channels to transmitter release.

Under experimental conditions where these variables have been somewhat controlled and are comparable to conditions employed at the developing calyx of Held synapse, emerging evidence suggests that several conventional mammalian central synapses employ nanodomain Ca^2+^ signaling (Eggermann et al., 2011). For example, nanodomain Ca^2+^ signaling is found both at hippocampal basket cell-granule cell inhibitory synapses and at cerebellar parallel fiber-Purkinje cell excitatory synapses (Bucurenciu et al., 2008; Eggermann and Jonas, 2011; Schmidt et al., 2013). In addition, transmission at sensory synapses other than the calyx of Held apparently employ nanodomain coupling (Brandt et al., 2005; Jarsky et al., 2010). Together, results from a wide variety of mammalian synapses support the notion that nanodomain coupling is highly conserved and is therefore a general scheme for Ca^2+^ triggering of rapid neurotransmitter release.

## Conclusions

We have highlighted key evidence from two giant synapses, the squid giant synapse and the calyx of Held synapse, that firmly establish nanodomains as the predominant mode of Ca^2+^ signaling that couples Ca^2+^ influx through presynaptic Ca^2+^ channels to vesicular fusion and synaptic transmission. There is no doubt that nanodomains exist at many synapses, but it is interesting to note that this mode of Ca^2+^ signaling appears to be primarily associated with synapses for fast-spiking neurons, where synaptic transmission is particularly important for preserving temporal precision of information flow (Yang et al., 2014). Nanodomains with tight Ca^2+^ signaling may be particularly suited to trigger highly synchronized fusion events with minimal synaptic delay, temporal jitter, or asynchronous transmitter release. The narrow action potentials typical of fast-spiking neurons are likely to produce brief Ca^2+^ signals with rapid rise and collapse of local [Ca^2+^]. Brief Ca^2+^ transients will also serve to prevent presynaptic Ca^2+^ overload and facilitate Ca^2+^ clearance during prolonged high-frequency transmission. On the other hand, microdomains with loose coupling between Ca^2+^ channels and Ca^2+^ sensors may allow a broader dynamic range for tuning quantal output at synapses where activity-dependent forms of synaptic plasticity play a key role in dynamically regulating synaptic efficacy (Vyleta and Jonas, 2014).

Given the dimensions of SV and active zones, it is reasonable to postulate that the nanodomain Ca^2+^ signaling requires specific molecular specializations to optimize physical interactions between Ca^2+^ channels and SV (Catterall and Few, 2008; Südhof, 2013). To advance our understanding of local Ca^2+^ signaling in presynaptic terminals, it will next be important to define the specific molecular specializations underlying both microdomain and nanodomain Ca^2+^ signaling, and how these two modes of Ca^2+^ signaling are regulated during development. Emerging evidence indicate that several presynaptic proteins help tether Ca^2+^ channels to SV; these proteins include RIM (Kiyonaka et al., 2007; Kaeser et al., 2011), Munc 13 (Chen et al., 2013), bassoon (Davydova et al., 2014) and even synaptotagmin (Young and Neher, 2009). Genetic deletion of the cytometrix protein Bruchpilot loosens the coupling of Ca^2+^ channels and sensors at *Drosophila* presynaptic terminals (Kittel et al., 2006). In contrast, knockout of septin 5, a filamentous protein that interacts with the SNARE proteins that mediate exocytosis, transforms microdomain coupling typical of immature synapses to nanodomain coupling with functional and morphological phenotypes nearly identical to mature calyces (Figure 6C; Yang et al., 2010). These results suggest that these two modes of local Ca^2+^ signaling are differentiated by specific molecular substrates and potentially could be inter-convertible to confer distinctive Ca^2+^ signaling properties to synapses.

## Conflict of interest statement

The authors declare that the research was conducted in the absence of any commercial or financial relationships that could be construed as a potential conflict of interest.
